# Knockdown of nuclear receptor binding SET domain-containing protein 1 (NSD1) inhibits proliferation and facilitates apoptosis in paclitaxel-resistant breast cancer cells via inactivating the Wnt/β-catenin signaling pathway

**DOI:** 10.1080/21655979.2021.2018973

**Published:** 2022-02-24

**Authors:** Yi Chen, Xiao Li, Jin Xu, Hua Xiao, Cuiju Tang, Wei Liang, Xuedan Zhu, Yueyu Fang, Hanjin Wang, Junfeng Shi

**Affiliations:** a Department of Oncology, Nanjing Pukou Central Hospital, The First Affiliated Hospital of Nanjing Medical University, Nanjing, Jiangsu, China; b Department of Thyroid and Mammary Gland Surgery, Nanjing First Hospital, Nanjing Medical University, Nanjing, Jiangsu, China; c Department of General Surgery, Nanjing First Hospital, Nanjing Medical University, Nanjing, Jiangsu, China; d Department of Oncology, Nanjing First Hospital, Nanjing Medical University, Nanjing, Jiangsu, China

**Keywords:** BC, NSD1, wnt/β-catenin signaling pathway, H3K27me3, Wnt10b

## Abstract

The burden of breast cancer (BC) has exacerbated over decades. Paclitaxel resistance is responsible for increasing BC treatment burden. Nuclear receptor binding SET domain-containing protein 1 (NSD1) is positively correlated with a poor prognosis in patients with BC. This study investigates the function of NSD1 in paclitaxel-resistant (PR) BC cells. The high levels of NSD1 and Wnt10b in PR BC cell lines (MCF-7/PR) or MCF-7 parental cells were determined by RT-qPCR. Western blotting was conducted to measure the levels of NSD1 protein, apoptosis-associated proteins, Wnt10b protein, H3K36me2 protein, H3K27me3 protein, and signal pathway-associated proteins in MCF-7/PR cells or MCF-7 cells or *in vivo* subcutaneous xenografted tumor model, and the results demonstrated that NSD1 inhibited cell apoptosis and promoted cell proliferation and tumor growth via activating Wnt/β-catenin pathway. Cell apoptosis and viability were estimated using cell counting kit-8 assays and flow cytometry. Positive correlation between NSD1 and Wnt10b was identified by chromatin immunoprecipitation assay. The distribution of β-catenin was determined by immunofluorescence assays. We conclude that NSD1 knockdown inhibits the viability and promotes the apoptosis of paclitaxel-resistant BC cells by inactivating the NSD1/H3K27me3/Wnt10b/β-catenin signaling pathway.

## Introduction

Breast cancer (BC) presents a threatening risk for female and the second-leading malignancy causing death for female [1,2]. BC made up of 24.2% of all female cancer cases in 2018 and attributed to 15% of female cancer deaths [3]. Currently, surgery, chemotherapy, and radiation are the three most common treatments for breast cancer [4]. Despite great advancements, the morbidity and mortality of BC are still increasing [5]. Recurrence and metastasis after treatment pose severe threats to survival [6]. Chemotherapy is the most basic strategy among these therapies [7]. However, drug resistance and blocked drug delivery lead to the failure of treatment in many BC cases [8,9]. Paclitaxel, an effective chemotherapeutic drug to accelerate cancer cell apoptosis by exerting impacts on microtubule stabilization, is widely accepted for BC treatment [10–12]. However, paclitaxel resistance is a major cause of treatment failure. Wnt/β-catenin signaling is associated with the drug resistance in tumor treatment, including BC treatment, squamous cell carcinoma treatment, and ovarian cancer treatment [13–18]. Wingless-related mouse mammary tumor virus integration site 10b (Wnt10b), a member of the Wnt ligand gene family induces the Wnt/β-catenin signaling pathway activation [19]. Thus, we investigated the regulatory mechanisms underlying the paclitaxel resistance in BC via Wnt/β-catenin signaling.

Nuclear receptor binding SET domain (NSD) proteins consist of NSD1, NSD2, and NSD3. These proteins participate in the regulation of tumor initiation and progression [20]. Increasing evidence has proven that NSD family is involved in BC initiation and progression. For example, NSD3, located on the Wolf-Hirschhorn syndrome candidate 1-like 1 (WHSC1L1) gene, is upregulated in BC cell lines, facilitating the progression of BC [21]. Higher NSD2 expression in BC cell lines contributes to cancer recurrence and unfavorable prognosis [22]. However, the biological functions of NSD1 in BC progression remains unclear. As reported, NSD1 is a potential target for cancer diagnosis and treatment. For example, hypermethylation mediated by NSD1 is related to a poor prognosis in patients with high-risk neuroblastoma [23]. Deregulation of DNA methylation induced by NSD1 silencing in head and neck squamous cell carcinoma indicates a good prognosis [24]. The result of Kaplan Meier survival curves indicate that NSD1 upregulation is positively correlated with unfavorable prognosis. Moreover, NSD1 is reported to regulate hepatocellular carcinoma progression through the Wnt/β-catenin signaling pathway [19].

We here purposed to explore the underlying mechanisms and biological functions of NSD1 in BC progression. We formulated a hypothesis that NSD1 would strengthen BC cell drug resistance and lead to poor prognosis in patients with BC. Our study will offer theoretical support for BC treatment.

## Methods

### Tissue specimens

Breast tumor tissue samples (N = 63) and normal adjacent samples (N = 63) were obtained from 63 patients with BC who underwent surgeries at Nanjing First Hospital, Nanjing Medical University (Jiangsu, China). Written informed consent was offered by patients. The study was permitted by the ethics committee of Nanjing First Hospital, Nanjing Medical University (Jiangsu, China). Liquid nitrogen was used to freeze tissue samples. The frozen samples were then reserved at −80°C.

### Cell culture

The human BC cell line MCF-7 was obtained from COBIOER (Nanjing, China) and incubated in a paclitaxel for over 6 months, and we gradually increased the paclitaxel concentration[4]. Thereafter, MCF-7/PR cell line was established. The DMEM medium (Gibco, Waltham, CA, USA) supplemented with 10% fetal bovine serum (FBS; Gibco) containing 100 mg/mL streptomycin (Sigma-Aldrich, St-Louis, MO, USA) and 100 U/mL penicillin (Sigma-Aldrich) was used to incubate cells. Cell incubation was under a humid condition containing 5% CO_2_ at 37°C.

### Lentiviral transduction and cell transfection

The lentiviral transduction and cell transfection were performed as previously described [25]. NSD1 small interfering RNA (si-NSD1) and its negative control (si-NC) were constructed into lentiviruses to stably knockdown NSD1. The sequences of si-NSD1 are 5ʹ-GGAACUUCAUCAUCAUCUACUUAGAUGAUGAUGAAGUUCCAG-3ʹ. The lentiviral vectors containing full length of NSD1 (LV-NSD1) were used to overexpress NSD1 with empty lentiviral vector-negative control (LV-NC). All lentiviral vectors were obtained from GenePharma (Shanghai, China). The MCF-7 and MCF7/PR cell lines were transfected with these vectors packaged with an inactivated human immunodeficiency virus using Polybrene (Yeasen, Shanghai, China). After 48 h, RT-qPCR was used to measure transfection efficiency. The experiments were repeated three times.

### Reverse transcription quantitative polymerase chain reaction (RT-qPCR)

RT-qPCR was conducted under the instruction of a previous study [26]. The total RNA was extracted from MCF-7 and MCF-7/PR cells using TRIzol reagent (Invitrogen, Carlsbad, CA, USA) and reverse transcribed into cDNA using Reverse Transcription Kit (Takara Bio, Beijing, China). The ABI7500 system (Applied Biosystem, Foster City, CA, USA) was used to perform RT-qPCR analysis. GAPDH served as an internal reference. The 2^−ΔΔCt^ method was used to analyze the relative expression. The primer sequences are listed in Table 1. The experiments were performed three times.

**Table 1.** Sequences of primers used for reverse transcription-quantitative PCR

Gene	Sequence (5ʹ→3ʹ)
NSD1 forward	GTGACATAGAAACAGCAGTGGTGA
NSD1 reverse	GATGGCTTTGATGTTCCAGAG
Wnt10b forward	GAATGCGAATCCACAACAACAG
Wnt10b reverse	TTGCGGTTGTGGGTATCAATGAA
GAPDH forward	AATCCCATCACCATCTTCCA
GAPDH reverse	TGGACTCCACGACGTACTCA
U6 forward	GCTTCGGCAGCACATATACTAAAAT
U6 reverse	CGCTTCACGAATTTGCGTGTCAT

### Western blotting

Western blotting was conducted to measure the protein levels in MCF-7 cell lines and tumor tissues, as previously reported [27]. A total of 4 pairs of tumor and nontumor tissues from patients with BC were collected unintentionally. Proteins were separated using a RIPA buffer (Sigma-Aldrich) and a protease inhibitor (Sigma-Aldrich). The protein concentrations in the lysates were measured with the Protein BCA Assay Kit (Sigma-Aldrich). Protein samples (20 µg) were resolved in sodium dodecyl sulfate-polyacrylamide gel electrophoresis (SDS-PAGE) gels (Thermo Fisher Scientific, Waltham, MA, USA), and resolved proteins were transferred onto the polyvinylidene fluoride membranes (BioRad, Hercules, CA, USA). Five percent skimmed milk was used to block the membranes at room temperature. After 1 h, the membranes were incubated with primary antibodies against NSD1 (abx432373, 0.5 mg/mL; Abbexa), β-actin (ab8227, 1:1000; Abcam), Ki67 (ab92742, 1:5000; Abcam), PCNA (ab92552, 1:1000; Abcam), cleaved caspase-3 (ab32042, 1:500; Abcam), cleaved caspase-9 (ab2324, 1 µg/mL; Abcam), Wnt10b (ab70816, 2 µg/mL; Abcam), H3K36me2 (abx000012, 1:1000; Abbexa), H3K27me3 (abx000010, 1:1000; Abbexa), H3 (ab1791, 1:1000; Abcam), β-catenin (ab16051, 0.25 µg/mL; Abcam), cyclin D1 (ab226997, 1:1000; Abcam), and c-Myc (ab32072, 1:1000; Abcam) overnight at 4°C. Phosphate buffered saline (PBS; Solarbio, Beijing, China) was used to wash the membranes. After incubation for 1 h with secondary antibodies at room temperature, the protein bands were observed with Luminol Reagent (Sigma-Aldrich), and the intensity was assessed using ImageJ software (National Institutes of Health, Bethesda, MD, USA). β-actin was used as a loading control. The experiments were conducted three times.

### Cell counting kit-8 (CCK-8) assays

CCK-8 assays were conducted to assess cell viability in accordance with a previous method [26]. Cells were seeded into 96-well plates (1 × 10^3^ cells/well) after transfection. At 0 h, 24 h, 48 h, and 72 h, CCK-8 reagent (10 μL; Sigma-Aldrich) was subsequently added. After incubation for 2 h, a microscope reader (Thermo Fisher Scientific) was utilized to evaluate the optical value at 450 nm. This experiment was repeated three times.

### Apoptosis analysis

Cell apoptosis was estimated as previously reported [28]. After transfection, the cells were stained with propidine iodide and Annexin V labeled by fluorescein isothiocyanate (Abcam, Cambridge, MA, USA) at 4°C in the darkness for 15 min. The FACSCalibur^TM^ System (BD Biosciences, NY, New York, USA) was utilized to determine cell apoptosis. The experiments were carried out three times.

### Chromatin immunoprecipitation (CHIP) assay

CHIP assays were performed according to a previous protocol [29]. After 48 h transfection, 1% polyformaldehyde solution (Sigma-Aldrich) was used to fix the cells and glycine stock solution (Cayman, Ann Arbor, Michigan, USA) was utilized to quench the cells. Then, cold PBS (Solarbio) was used to wash the cells. After washing, the cells were centrifuged at 2000 rpm for 5 min and then suspended in SDS lysis buffer (GBCBIO, Guangzhou, China). After incubation on ice for 10 min, the pellets were ultrasonicated to obtain chromatin fragments. ChIP dilution buffer (Lab EAD, Beijing, China) containing a protease inhibitor was then utilized to dilute the supernatant. Thereafter, the blocking solution was added, and the incubation was kept at 4°C for 30 min. A small part of the supernatant was taken as input and the remaining supernatant was incubated with antibody against H3K27me3 (abx000010, 1:1000; Abbexa) and anti-rabbit IgG (ab6721, 1:2000; Abcam) in the negative control group overnight at 4°C. The supernatant was removed after 700 rpm centrifugation for 1 min, and the precipitants were washed with an elution buffer. Protein K digestion (Thermo Fisher Scientific) was conducted to remove protein, and DNA was purified by the Min-Elute PCR purification kit (Qiagen, Nasdaq, NY, USA). The relative expression of H3K27me3 was measured by fluorescence qPCR. This assay was repeated three times.

### Immunofluorescence assay

Immunofluorescence assays were conducted as previously described [30]. After incubation in 96-well plates for 24 h, the cells were fixed with 4% paraformaldehyde and permeabilized with 0.2% Triton X-100 for 15 min. Then, the cells were blocked from nonspecific bindings using 1% bovine serum albumin in PBS (Solarbio) for 1 h. Next, primary antibodies against β-catenin (ab16051, 0.25 µg/ml; Abcam) were added. After incubation overnight at 4°C, cells were sufficiently washed. Then, fluorescence secondary antibodies (ab150077, 1:1000; Abcam) were added. After 30 min, nuclei were stained using DAPI (Sigma-Aldrich) for 10 min. The images were taken using laser confocal microscopy (Carl Zeiss Co. Ltd., Jena, Germany). The experiment was performed three times.

### A xenograft tumor model

Animal experiments were conducted under the permission of the Institutional Animal Care and Use Committee of Nanjing First Hospital, Nanjing Medical University (Jiangsu, China). The experimental procedures conformed with the guidelines of the National Institutes of Health. Six male BALB/c-nude mice (4 weeks old, 20 g) were obtained from Jiangsu ALF Biotechnology Co., LTD (Nanjing, China), and these mice were bred in a specific-pathogen-free environment. Mice were divided into the si-NSD1 group and si-NC group, and each group had three mice. The 5 × 10^6^ MCF-7 cells transfected with lentivirus wrapped si-NSD1 or si-NC were injected subcutaneously into the back of nude mice. The tumor diameter was evaluated every 7 days for 5 weeks. Five weeks later, all the mice were sacrificed. The mice tumors were separated precisely from the adjacent normal structures. The tumor weight was weighed, and the volume was estimated using the formula: volume = (width^2^ × length)/2.

### Statistical analysis

SPSS 20.0 (Chicago, IL, USA) and Graphpad Prism 6.0 were used to analyze statistics. Data are presented as mean ± standard deviation. *p* < 0.05 was considered statistically significant. Student’s *t* test was used for two groups of comparisons and one-way analysis of variance with Turkey’s *post hoc* analysis was conducted for comparisons of multiple groups.

## Results

Bioinformatics analysis indicates a positive correlation between high NSD1 level and poor prognosis in patients with BC. NSD1 has been reported to serve as a regulator for various cancers, such as head and neck cancer, pancreatic cancer, and hepatocellular cancer. Thus, we conducted a series of in vitro and in vivo experiments to investigate the biological functions and underlying mechanisms of NSD1 in CC progression. We found that NSD1 knockdown inhibits the viability and promotes the apoptosis of paclitaxel-resistant BC cells by inactivating the NSD1/H3K27me3/Wnt10b/β-catenin signaling pathway.

### NSD1 level is upregulated in BC tissues and cells

**Figure 1.**
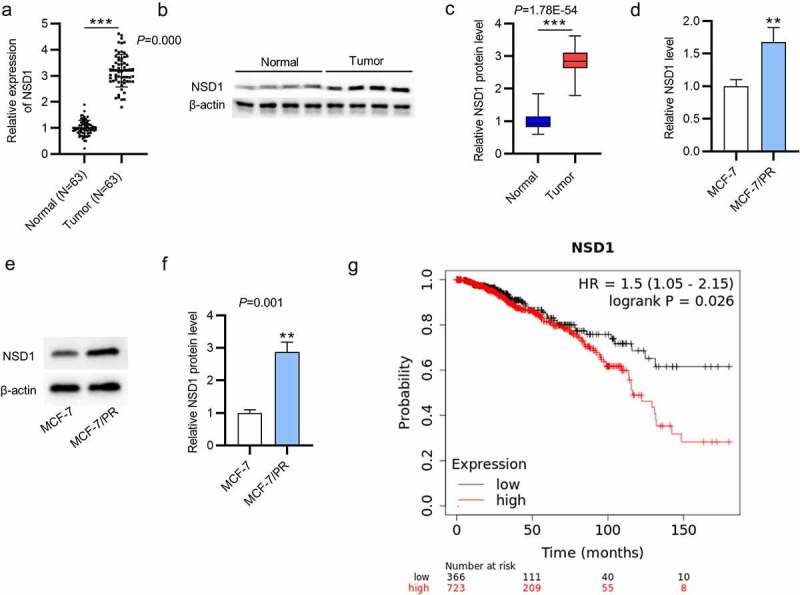
NSD1 is upregulated in BC tissues and cells. (a-c) RT-qPCR and Western blotting of NSD1 mRNA and protein levels in BC tissues. (d-f) RT-qPCR and Western blotting of NSD1 mRNA and protein levels in MCF-7 and MCF-7/PR cells. (g) Kaplan-Meier (https://kmplot.com) survival curves of the correlation between NSD1 expression and patient prognosis. **p < 0.01, ***p < 0.001.

To explore the biological functions of NSD1 in BC progression, RT-qPCR and Western blotting were conducted to measure NSD1 expression in BC tissues and cells. The NSD1 mRNA level was upregulated in BC tissue samples compared with those in normal tissue samples (p = 0.000), and the protein level of NSD1 was also increased in BC tissue samples (p = 1.78E^−54^) (Figure 1(a-c)). We conducted the same experiments to measure its levels in BC cells. The mRNA and protein levels of NSD1 were higher in MCF-7/PR cells than in parental MCF-7 cells (Figure 1(d-f)). Kaplan-Meier (https://kmplot.com) survival curves indicate that high NSD1 level is positively related to unfavorable prognosis in patients with BC (Figure 1(g)). We conclude that NSD1 is upregulated in BC tissues and cells.

### NSD1 promotes the proliferation and inhibits the apoptosis of BC cells

**Figure 2.**
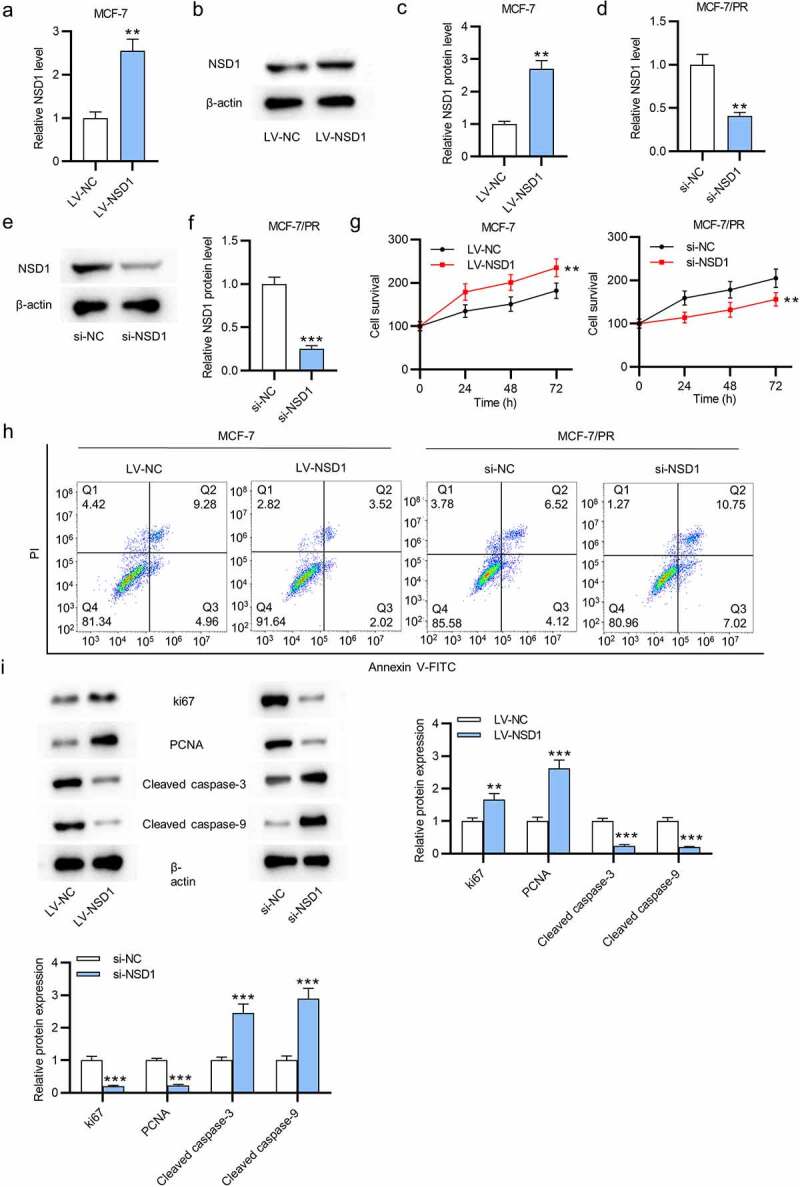
NSD1 promotes the proliferation and inhibits the apoptosis of BC cells. (a-c) RT-qPCR and Western blotting estimated the overexpression efficiency of LV-NSD1 in MCF-7 cells. (d-f) RT-qPCR and Western blotting investigated the downregulation efficiency of si-NSD1 in MCF-7/PR cells. (g) CCK-8 assays of cell viability in MCF-7 cells and MCF-7/PR cells after indicated transfection. (h) Flow cytometry of cell apoptosis after transfection. (i) Western blotting was conducted to measure the levels of proteins associated with cell proliferation and apoptosis. **p < 0.01, ***p < 0.001.

Considering the relatively low expression of NSD1 in MCF-7 cells, we effectively overexpressed it using LV-NSD1. As shown by RT-qPCR and Western blotting, LV-NSD1 effectively upregulated NSD1 level in MCF-7 cells (Figure a-c). On the contrary, we effectively knocked down the NSD1 level in MCF-7/PR cells using si-NSD1 (Figure d-f). To investigate the biological functions of NSD1, we conducted CCK-8 assays to estimate cell proliferation in response to the abnormal expression of NSD1. The results revealed that NSD1 upregulation promoted the proliferative abilities of MCF-7 cells, while NSD1 silencing inhibited cell proliferation of MCF-7/PR cells (Figure 2(g)). The results of flow cytometry demonstrated that NSD1 elevation inhibited the apoptosis of MCF-7 cells, while NSD1 depletion enhanced the apoptosis of MCF-7/PR cells (Figure 2(h)). Finally, as shown by Western blotting, NSD1 upregulation increased the levels of Ki67 and PCNA proteins that were associated with cell proliferation and decreased the levels of cleaved caspase- and cleaved caspase-9 proteins that were related to cell apoptosis in MCF-7 cells, while NSD1 downregulation had the opposite effect in MCF-7/PR cells (Figure 2(i)). In summary, NSD1 facilitates the proliferation and inhibits the apoptosis of BC cells.

### NSD1 positively regulates Wnt10b expression

**Figure 3.**
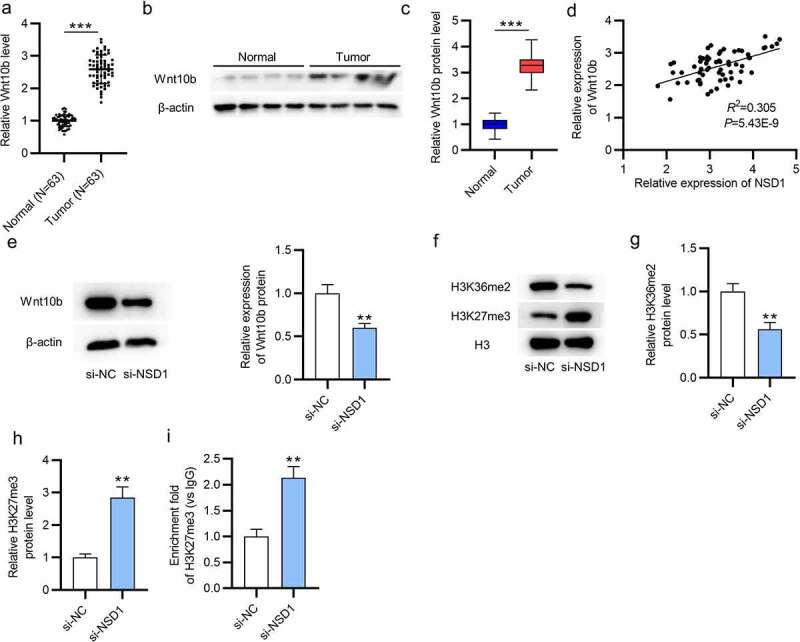
NSD1 level is positively correlated with Wnt10b level. (a-c) RT-qPCR and Western blotting of Wnt10b mRNA and protein levels in BC tissues. (d) GEPIA (http://gepia.cancer-pku.cn/detail.php) reveals the correlation between NSD1 expression and Wnt10b expression in BC tissues. (e) Western blotting of the Wnt10b protein level after transfection of si-NC or si-NSD1. (f-h) Western blotting assessed the levels of methylation-associated proteins after transfection. (i) CHIP assay of the H3K27me3 enrichment in the Wnt10b promoter. **p < 0.01, ***p < 0.001.

As shown by RT-qPCR and Western blotting, the mRNA, and protein levels of Wnt10b in BC tissues (N = 63) were higher than those in normal tissues (N = 63) (Figure 3(a-c)). Then, we discovered the positive correlation between NSD1 and Wnt10b level in BC tissues using GEPIA (p = 5.43E^−9^) (Figure 3(d)). The protein level of Wnt10b was reduced in response to NSD1 knockdown, as shown by Western blotting (Figure 3(e)). As reported, H3K27me3 enrichment in the Wnt10b promoter region inactivates the Wnt/β -catenin signaling pathway [31,32]. Thus, we measured H3K27me3 expression in BC cells to detect the correlation between NSD1 and Wnt10b. The results showed that NSD1 depletion elevated the protein level of H3K27me3 and opposingly downregulated the protein level of H3K36me2 (Figure F-H). Additionally, NSD1 silencing increased the H3K27me3 enrichment in the Wnt10b promoter region, as shown by CHIP assays (Figure 3(i)). In summary, the NSD1 knockdown in BC cells facilitates H3K27me3 enrichment in Wnt10b promoter, leading to the downregulation of Wnt10b.

### NSD1 knockdown inactivates the Wnt/β-catenin signaling pathway

**Figure 4.**
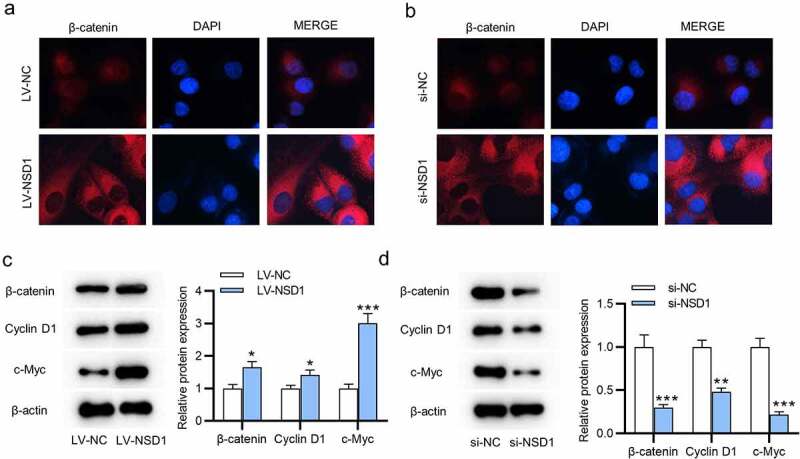
NSD1 knockdown inactivates Wnt/β-catenin pathway. (a-b) Immunofluorescence assays identified the distribution of β-catenin after transfection of LV-NC or LV-NSD1 and si-NC or si-NSD1. (c-d) Western blotting detected the levels of proteins that related to the Wnt/β-catenin signaling pathway after transfection. *p < 0.05, **p < 0.01, ***p < 0.001.

To investigate NSD1 functions on the Wnt/β-catenin signal pathway, we conducted immunofluorescence assays. The results reveal that NSD1 upregulation facilitates the nuclear translocation of β-catenin, while NSD1 knockdown causes the cytoplasmic distribution of β-catenin, suggesting that NSD1 facilitated the Wnt/β-catenin signaling pathway activation (Figure 4(a,b)). Then, the levels of β-catenin, cyclin D1, c-Myc proteins were increased after NSD1 overexpression, and NSD1 downregulation had an opposite effect, as revealed by Western blotting (Figure 4(c,d)).

### NSD1 knockdown inhibits the tumor growth in vivo

**Figure 5.**
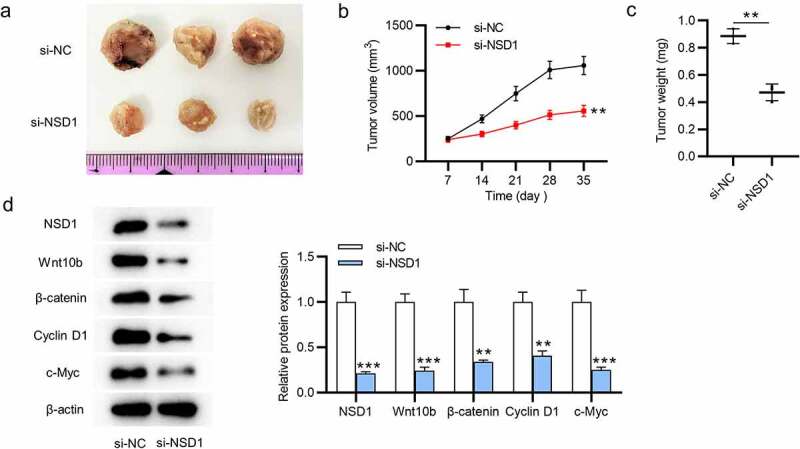
NSD1 silencing suppresses tumor growth *in vivo*. (a-c) A xenograft model was established to detect tumor growth after injection of MCF-7 cells transfected with lentivirus wrapped si-NC or si-NSD1 *in vivo*. Tumor image, tumor volume curves and tumor weight were presented. (d) Western blotting evaluated the levels of the proteins that related to the Wnt/β-catenin signaling pathway. **p < 0.01, ***p < 0.001.

The biological functions of NSD1 *in vivo* were determined by animal experiments. We found reduced tumor volumes and decreased tumor weight after injection of MCF-7 cells transfected with NSD1 silencing (Figure 5(a-c)). The levels of NSD1, Wnt10b, β-catenin, cyclin D1, c-Myc proteins that are associated with Wnt/β-catenin signaling pathway were decreased, as Western blotting showed (Figure 5(d)). In summary, NSD1 silencing inhibits tumor growth by inhibiting the Wnt/β-catenin signaling pathway activation.

## Discussion

BC, a female-common cancer, is characterized with a high incidence and mortality [33]. The NSD family is upregulated in various cancers, as reported [34]. NSD1, one of the paralogous proteins of NSD family, plays essential roles in tumorigenesis [35]. The high NSD1 expression in hepatocellular carcinoma promotes cellular processes [19]. Additionally, NSD1 results in hypermethylation, which brings about poor prognosis in patients with neuroblastoma [23]. Moreover, for head and neck squamous cell carcinoma, NSD1 knockdown promotes the apoptosis of inflammation cytokines and leads to the deregulation of DNA methylation [24,36]. We herein discovered that the NDS1 was upregulated in BC tissues and cells. Additionally, NSD1 enhanced proliferation and repressed destruction of BC cells. Moreover, *in vivo* experiments suggested that NSD1 silencing repressed tumor growth.

The Wnt/β-catenin signaling pathway is involved in cancer malignancies, including cellular processes and drug resistance [37–41]. As reported, increased H3K27 methylation in the Wnt10b promoter restrains its transcriptional ability, thereby inactivating the Wnt/β-catenin signaling pathway [32]. Wnt10b activates the Wnt/β-catenin signaling pathway and exerts functions in various malignancies, including gastric cancer, colorectal cancer, cervical cancer [42–45]. Herein, we discovered a positive correlation between NSD1 and Wnt10b in BC samples. We further investigate the underlying mechanism of NSD1 regulating Wnt10b. Results indicate that NSD1 knockdown facilitated H3K27me3 enrichment in the Wnt10b promoter, thereby inactivating the Wnt/β-catenin signaling in BC.

β-catenin, a polyfunctional protein, exerts inevitable effects on various cancers initiation and progression, such as colon cancer, hepatocellular cancer, and oral cancer [46–48]. Wnt is proved to regulate β-catenin expression that Wnt ligands absence results in β-catenin downregulation [49]. Similarly, we herein discovered that the LV-NSD1-mediated increased Wnt10b level facilitated β-catenin to accumulate in the nucleus, thereby promoting the upregulation of oncogenes, including c-Myc and Cyclin D1, while si-NSD1 had an opposite result. Therefore, the Wnt/β-catenin signaling pathway was inactivated after NSD1 knockdown in BC.

However, our study also has some limitations. First, the relationship between NSD1 and prognosis in patients with BC is short of practical proof in our study. Second, the mechanism underlying NSD1 knockdown regulating H3K27me3/Wnt10b/β-catenin in BC remains inadequately investigated.

## Conclusion

In summary, high expression of NSD1 was observed in BC tissues and cells, and NSD1 silencing inhibited proliferation and promoted apoptosis by inactivating the Wnt/β-catenin signaling pathway. NSD1 would be a promising target for BC diagnosis and treatment.

## Data Availability

We declare that the data that support the findings of this study are available from the corresponding author upon reasonable request.
